# The quest for an unbiased scientific impact indicator remains open

**DOI:** 10.1073/pnas.2410021121

**Published:** 2024-09-30

**Authors:** Giacomo Vaccario, Shuqi Xu, Manuel S. Mariani, Matúš Medo

**Affiliations:** ^a^Chair of Systems Design, Department of Management, Technology, and Economics, ETH Zürich, Zürich CH-8006, Switzerland; ^b^Institute of Dataspace, Comprehensive National Science Center, Hefei 230088, People’s Republic of China; ^c^University Research Priority Program Social Networks, Department of Business Administration, University of Zurich, Zurich CH-8050, Switzerland; ^d^Department for BioMedical Research, Inselspital, Bern University Hospital, University of Bern, Bern CH-3008, Switzerland; ^e^Department of Radiation Oncology, Inselspital, Bern University Hospital, University of Bern, Bern CH-3008, Switzerland

Developing unbiased indicators of scientific impact has long been a central question in the scientometrics and science of science communities (1, 2). Ke et al. (3) recently tackled the ambitious challenge of developing a paper-level network-based indicator that can be fairly compared across time and fields even without the need for a field classification system, concluding that their proposed ${\hat{c}}_{10}$ achieves this objective. The idea of leveraging a network-based mechanism to prevent impact indicator bias provides a compelling perspective to the long-standing debate on indicator bias, which could inspire many future works. Unfortunately, the validation performed in the paper does not properly test for bias, nor does it test properly for the indicator’s ability to detect groundbreaking research.

**Fig. 1.**
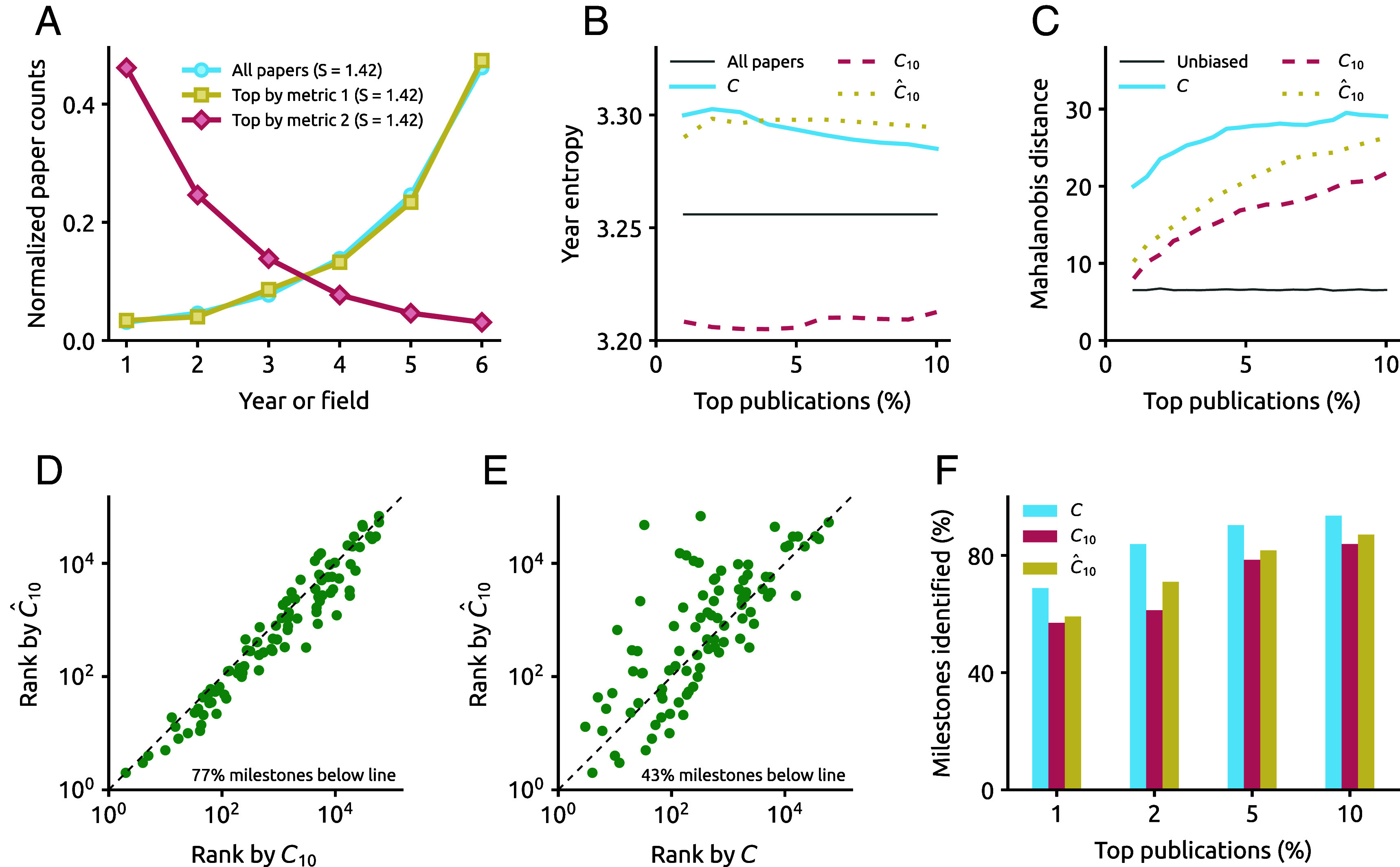
(*A*) Illustrative year distributions of two hypothetical metrics and the whole corpus of papers, all with similar Year entropy S. (*B*) The Year entropy for the top papers obtained by three evaluated metrics and for all papers in APS data (7). (*C*) The Mahalanobis distance of the top papers obtained for the evaluated metrics and the 95th percentile of the Mahalanobis distance obtained for simulated unbiased distributions (6). (*D*) Ranking positions of the 87 PRL milestone letters (8) by ${\hat{c}}_{10}$ and $c_{10}$ (citation count 10 years after publication), as in figure 3C in ref. 3. (*E*) Ranking positions of the PRL milestone letters by ${\hat{c}}_{10}$ and c (raw citation count). (*F*) The fraction of PRL milestones among the top publications (8) for the evaluated metrics.

To test for age bias, ref. 3 considers an indicator’s top p% publications and measures their diversity in time using the entropy of the normalized year count distribution. Interpreting entropy as a proxy for bias is meaningful only if the unbiased distribution is uniform. However, for all papers to have an equal chance of being top-ranked, the unbiased distribution is the papers’ yearly distribution, which is strongly uneven (4). Fig. 1*A* shows a nearly unbiased metric 1 and a strongly biased metric 2. However, Year entropy suggests that both metrics have the same bias level, thus defeating the original purpose of the bias test.

A more appropriate test for bias relies on measuring the distance between the year distribution of the top p% papers by a given metric and that of all papers (hereafter, unbiased distribution) (5, 6). In the American Physical Society (APS) citation dataset (7), Year entropy values are in the same range as reported in ref. 3 (Fig. 1*B*). The distance-based test (6) reveals that ${\hat{c}}_{10}$ nevertheless exhibits a strong age bias. Its Mahalanobis distance (6) from the distribution of all papers is higher than the 95th percentile of the distances achieved by simulated unbiased distributions and it is even more biased than $c_{10}$ (Fig. 1*C*). We find that this is due to ${\hat{c}}_{10}$’s bias toward older papers, already evident in figure 4C in ref. 3.

To validate ${\hat{c}}_{10}$’s ability to identify groundbreaking research, ref. 3 shows that most APS milestone letters are better ranked by ${\hat{c}}_{10}$ than by $c_{10}$. We replicate their result in the APS dataset (Fig. 1*D*). However, raw citation count c outperforms both $c_{10}$ and ${\hat{c}}_{10}$ (Fig. 1 *E* and *F*). The reason is that, unlike previous works (7, 8), the temporal distribution of the milestones is not controlled for in ref. 3. As raw citation count c favors old papers and the APS milestones tend to be old (7), ref. 3’s evaluation benefits c.

In sum, the idea of using network-based mechanisms to prevent biases is compelling, yet ${\hat{c}}_{10}$ is not unbiased. Beyond age and field, ranking systems can be subject to additional concerning biases (9), requiring even more caution when concluding indicators’ fairness. These nuances call for more research which could advance our understanding of the evolution of science as well as address broader issues on algorithmic bias and fairness.
